# Evaluating the impact of alcohol minimum unit pricing on deaths and hospitalisations in Scotland: a controlled interrupted time series study

**DOI:** 10.1016/S0140-6736(23)00497-X

**Published:** 2023-04-22

**Authors:** Grant M A Wyper, Daniel F Mackay, Catriona Fraser, Jim Lewsey, Mark Robinson, Clare Beeston, Lucie Giles

**Affiliations:** a Place and Wellbeing Directorate, Public Health Scotland, Glasgow, UK; b School of Health and Wellbeing, University of Glasgow, Glasgow, UK; c Institute for Social Science Research, University of Queensland, Brisbane, QLD, Australia

## Abstract

**Background:**

Since May 1, 2018, every alcoholic drink sold in Scotland has had minimum unit pricing (MUP) of £0·50 per unit. Previous studies have indicated that the introduction of this policy reduced alcohol sales by 3%. We aimed to assess whether this has led to reductions in alcohol-attributable deaths and hospitalisations.

**Methods:**

Study outcomes, wholly attributable to alcohol consumption, were defined using routinely collected data on deaths and hospitalisations. Controlled interrupted time series regression was used to assess the legislation's impact in Scotland, and any effect modification across demographic and socioeconomic deprivation groups. The pre-intervention time series ran from Jan 1, 2012, to April 30, 2018, and for 32 months after the policy was implemented (until Dec 31, 2020). Data from England, a part of the UK where the intervention was not implemented, were used to form a control group.

**Findings:**

MUP in Scotland was associated with a significant 13·4% reduction (95% CI –18·4 to –8·3; p=0·0004) in deaths wholly attributable to alcohol consumption. Hospitalisations wholly attributable to alcohol consumption decreased by 4·1% (–8·3 to 0·3; p=0·064). Effects were driven by significant improvements in chronic outcomes, particularly alcoholic liver disease. Furthermore, MUP legislation was associated with a reduction in deaths and hospitalisations wholly attributable to alcohol consumption in the four most socioeconomically deprived deciles in Scotland.

**Interpretation:**

The implementation of MUP legislation was associated with significant reductions in deaths, and reductions in hospitalisations, wholly attributable to alcohol consumption. The greatest improvements were in the four most socioeconomically deprived deciles, indicating that the policy is positively tackling deprivation-based inequalities in alcohol-attributable health harm.

**Funding:**

Scottish Government.

## Introduction

Harmful alcohol consumption is a leading risk factor of the global disease burden.^1^ In 2021, the highest number of deaths from alcohol-specific causes on record were reported for the UK.^2^ Within the UK, health harms from alcohol are disproportionately higher in Scotland, and are strongly patterned by level of socioeconomic deprivation to the extent that the death rate from alcohol-specific causes is over five times higher in the most, compared with least, deprived areas.^3^ This socioeconomic deprivation gradient remains after adjusting for alcohol consumption levels.^4^ In the wider public health context, over the last decade, there has been a slow-down in improvements in life expectancy, with evidence of increasing inequalities, potentially further worsened due to the COVID-19 pandemic and the ongoing cost-of-living crisis.^5^, ^6^, ^7^

As part of a comprehensive strategy to reduce levels of alcohol consumption and related harms, Scotland became one of very few countries in the world to implement minimum unit pricing (MUP), of £0·50 per unit, for alcoholic drinks sold directly to the public.^8^ The legislation was introduced on May 1, 2018. MUP sets a legal minimum price below which alcohol cannot be sold, and differs from taxation policies, which are potentially circumvented by retailer-based changes in alcohol pricing.^9^ Among the envisaged benefits of MUP are reduced deaths and decreased health-care use.^10^ As the heaviest drinkers typically drink the cheapest alcohol, MUP has the potential to positively tackle inequalities in health harms, and reduce harms in subgroups at greatest risk.^11^

Previous international modelling and observational studies have estimated the effect of increasing alcohol pricing as a mechanism to reduce alcohol-attributable harms.^12^, ^13^, ^14^, ^15^, ^16^, ^17^, ^18^, ^19^, ^20^ Some studies estimate that MUP can lead to reductions in consumption in the heaviest drinkers, a key pathway to reducing overall, and inequalities in, harm. In the Scottish context, following 3 years of MUP legislation, alcohol sales in Scotland were estimated to have reduced by 3%. This reduction was driven by reduced sales of spirits, cider, and perry through the off-trade (ie, the sale of alcohol for consumption off the premises, such as from supermarkets).^21^ Increases in average prices were higher in Scotland, compared with England and Wales, in the year following MUP being implemented.^22^ Whether the legislation led to reductions in alcohol-attributable deaths and hospitalisations at a population level is not known. Previous evidence has illustrated that immediate impacts on outcomes wholly attributable to alcohol are plausible, following changes in consumption.^23^

**Research in context**

**Evidence before this study**

Scotland has the highest levels of alcohol health harms in the UK, which are strongly patterned by level of socioeconomic deprivation to the extent that the death rate from alcohol-specific causes is over five times higher in the most, compared with least, deprived areas. As part of a comprehensive strategy to reduce levels of alcohol consumption and related harms, Scotland became one of very few countries in the world to implement minimum unit pricing (MUP), of £0·50 per unit, for alcoholic drinks sold directly to the public. Previous international modelling and observational studies have estimated the effect of increasing alcohol pricing as a mechanism to reduce alcohol-attributable harms. Some studies estimate that MUP can lead to reductions in consumption in the heaviest drinkers, a key pathway to reducing overall, and inequalities in, harm.

We identified literature from primary research studies that evaluated the impact of the implementation of alcohol MUP legislation on alcohol consumption, sales, or health outcomes, in Scotland. We searched PubMed for related papers published between May 2, 2018, and March 1, 2023, with the search terms (“alcohol” AND “minimum unit pricing” AND (“health” OR “consumption” OR “sales”) AND “Scotland”), with no language restrictions. Theoretical modelling studies using historical data to forecast outcomes were excluded, as were studies reporting subnational results. Several studies report that alcohol sales reduced in Scotland following the implementation of alcohol MUP legislation. Evidence following 3 years of implementation of alcohol MUP legislation indicates that population-level alcohol sales reduced by 3%, with the greatest reductions in alcohol purchasing observed in households that purchased the most alcohol before the policy was implemented. There was no causal evidence of country-level impact of the implementation of alcohol MUP legislation on deaths and hospital admissions wholly attributable to alcohol consumption in Scotland, before this study.

**Added value of this study**

We used high-quality data sources, pertaining to individual deaths and hospitalisations with recorded person-specific demographic attributes. Both UK countries under study have a universal health-care system free at the point of use; therefore, there was minimal risk of sampling or recruitment bias. The use of a controlled interrupted time series study design allowed us to infer that the estimated impacts were plausible causal effects attributable to the implementation of alcohol MUP legislation.

Our findings indicate that the implementation of alcohol MUP legislation in May, 2018 in Scotland led to significant reductions in deaths, and reductions in hospitalisations, wholly attributable to alcohol consumption. We evaluated effect modification of the legislation and found that reductions were largest in the 40% most socioeconomically deprived areas, and for males. These are groups that experience disproportionately high levels of alcohol health harms. This evidence from our study is consistent with the theory of change underpinning the legislation, and shows that alcohol MUP has reduced alcohol health harms.

**Implications of all the available evidence**

Previous evidence had indicated that the implementation of alcohol MUP reduced population-level alcohol sales by 3%. Other studies have found that the greatest reductions in alcohol purchasing were observed in households that purchased the most alcohol before the policy was implemented, with very little or no impact on those purchasing at lower levels. Our study illustrates how alcohol health harms have changed due to the implementation of this legislation, both overall and by population subgroup. Previous studies have indicated that the subgroups experiencing the greatest alcohol health harms tend to purchase the cheapest alcohol, and that most dose–response curves between alcohol consumption and adverse health outcomes show that the largest impacts could occur through shifts in consumption in the heaviest drinkers. Our findings indicate that the largest reductions in harms were in subgroups known to be experiencing disproportionately higher levels of alcohol health harms. In combination with the previous evidence on alcohol sales, our study suggests that a 3% reduction in population-level alcohol sales led to a 13% significant reduction in deaths, and 4% reduction in hospitalisations, wholly attributable to alcohol consumption.

The aim of this paper was to estimate the effect of alcohol MUP legislation on deaths and hospitalisations wholly attributable to alcohol consumption.

## Methods

### Study design

The study setting was Scotland, a country of the UK with a residential population of 5·5 million that implemented a policy legislating MUP for alcohol, where a single unit is equivalent to 10 mL or 8 g of pure alcohol. We used a controlled interrupted time series study design to estimate the impact of MUP on study outcomes, incorporating data from England, a part of the UK where the legislation was not implemented, to form a control group.^24^ Study outcomes were included for people aged 16 years and older. A summary of the study flow is presented in diagrammatic form (appendix p 3). We published a pre-specified statistical analysis plan before undertaking the study, and report minor changes to our pre-specified approach (appendix p 14).^25^ This study is reported in adherence with the STROBE statement (appendix p 15).^26^

### Study data and outcomes

For Scotland and England, we obtained routinely collected data on deaths and hospitalisations for causes wholly attributable to alcohol consumption before the legislation was implemented (Jan 1, 2012, to April 30, 2018) and 32 months thereafter (May 1, 2018, to Dec 31, 2020, inclusive).^3^, ^27^, ^28^, ^29^ Wholly attributable causes were defined using International Statistical Classification of Diseases and Related Health Problems 10th revision (ICD-10) codes, for each calendar month (appendix p 4). Deaths were defined using the underlying cause of death and registered date of death. Hospitalisations were defined using the main diagnosis and date of admission. Where multiple study outcomes were identified in a single month, the details of the first hospitalisation were chosen. Each study outcome was further defined by sex and age group (16–34 years, 35–64 years, and ≥65 years), using routinely collected individual-level characteristic data. Furthermore, we stratified study outcomes by socioeconomic deprivation group. This was achieved by linking each individual's postcode of residence to deprivation deciles based on small-level administrative geographies, as a proxy for individual-level socioeconomic status.^30^, ^31^ The Scottish Index of Multiple Deprivation was used to assign Scottish outcomes, and the Index of Multiple Deprivation was used to map English outcomes. These indices are commonly used in routine reporting of statistics from public health institutes, and are used to rank the distribution of within-country deprivation. Further information on study outcome definitions and allocation to deprivation deciles is published elsewhere.^25^

We sourced estimates of mid-year residential populations for each country by sex, age group, deprivation decile, and year.^32^, ^33^ Monthly populations were estimated using linear interpolation between mid-year population estimates. Additionally, we also sourced data on the level of COVID-19 restrictions imposed during the part of the study period that overlapped with the COVID-19 pandemic, separately for the governments of the UK and Scotland.^34^

### Statistical analysis

We estimated monthly rates per 100 000 residential population for each study outcome for each country, overall, and for each population subgroup. Monthly rates were adjusted to account for differences in month length. Monthly rates were decomposed into trend (the increasing or decreasing value) and seasonal (the monthly seasonal pattern) components using seasonal-trend decomposition LOESS methodology.^35^ When evidence of residual seasonality was present, adjustments were made to the seasonal window parameter until no patterning in the residuals remained.

We used controlled interrupted time series methods with seasonal autoregressive integrated moving average (SARIMA) errors to assess the effect of MUP legislation on study outcomes.^36^ Interrupted time series methods are appropriate to apply to outcomes consistently identified before, and after, an intervention of interest. Adjustments for underlying temporal and seasonal trends were incorporated.^37^ Models were also adjusted for the level of government restrictions imposed over time, and by country, during the COVID-19 pandemic, using the Oxford COVID-19 Government Response Tracker (OxCGRT).^34^ The OxCGRT is an index, ranging from 0 to 100, that incorporates systematic information on containment, economic, health system, and vaccine policy measures that governments implemented throughout the COVID-19 pandemic. Higher values indicate more stringent restrictions. Weighted averages were calculated using daily values so that the index value represented full monthly periods. The OxCGRT took a value of 0 for all months before the start of the pandemic.

We included a binary variable to reflect the implementation of MUP legislation in Scotland, taking a value of 0 from Jan 1, 2012, to April 30, 2018, and a value of 1 from May 1, 2018, to Dec 31, 2020 (inclusive). England was defined as the control group, where the legislation was not implemented.

We log-transformed rates and derived models for each study outcome for Scotland and England separately. We then used the control group data from England as a covariate in the Scottish SARIMA models to produce controlled models. Model effect estimates and 95% CIs were transformed to the absolute scale. A p-value cutoff of less than 0·05 was used to determine whether effect estimates were statistically significant for primary outcomes. Confidence intervals were not adjusted for multiplicity, so should not be used in place of hypothesis testing for subgroup analyses. To assess effect modification, we undertook exploratory analysis of study outcomes across a range of demographic and socioeconomic deprivation subgroups. The term significant is reserved to describe statistically significant results. When other effects have been observed that are not statistically significant, but might be of clinical or public health importance, we report the direction of the effect estimate. Further information on the methods used can be found in our pre-specified statistical analysis plan.^25^

EViews 13 software was used to undertake all time series decompositions, and all interrupted time series modelling was carried out using the econometrics toolbox from MATLAB 9.1 Update 2.

### Sensitivity analyses

We pre-specified several sensitivity analyses to test the robustness of study findings on primary study outcomes. These included: modelling the difference between the Scottish and English time series, instead of incorporating English data as a covariate in Scottish SARIMA models; using an unobserved components model to estimate the effect; removing the COVID-19 pandemic post-intervention period; using subnational regions of England as a control group as they were potentially more similar to Scotland; and adopting the use of a non-geographical control group.^38^ We selected genitourinary conditions as the non-geographical control group. This choice was made on the basis of ensuring that the choice of non-geographical control was not attributable to alcohol consumption; not expected to have been influenced by MUP; and was unlikely to have been displaced as a cause of death due to the emergence of COVID-19. We also did a falsification test by modelling the impact of MUP legislation as if MUP had been implemented 6 months before the actual intervention.

### Role of the funding source

The funder of the study had no role in study design, data collection, data analysis, data interpretation, or writing of the report.

## Results

**Figure 1:**
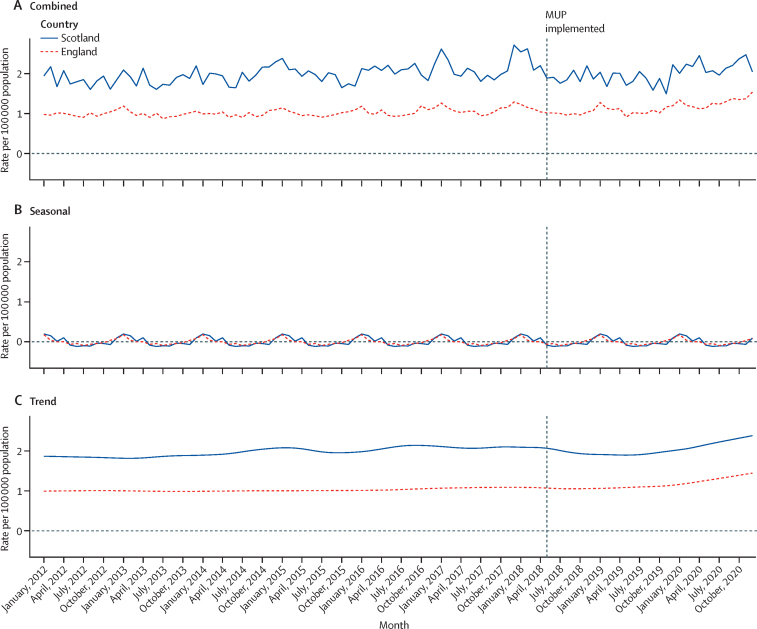
Rate of deaths wholly attributable to alcohol consumption per 100 000 population by country Monthly rate (A), and decomposed seasonal (B) and trend (C) components. MUP=minimum unit pricing.

The rate of deaths wholly attributable to alcohol consumption was relatively stable in both Scotland and England throughout the study period (figure 1). The underlying decomposed trend rate was approximately two deaths per month per 100 000 population in Scotland before the implementation of MUP legislation, approximately double the decomposed trend rate in England. Before the implementation of MUP legislation, there was a slight increase in the decomposed trend rate of deaths wholly attributable to alcohol consumption in Scotland, which decreased following the implementation of MUP legislation. From late 2019 to the end of the study period, the rate of deaths wholly attributable to alcohol consumption increased in both Scotland and England, and was the highest across the entire study period. Seasonality in the rate of deaths wholly attributable to alcohol consumption was evident in both Scotland and England, with rates peaking in January each year. The contribution of seasonality to the overall time series was stable throughout the full study period.

**Figure 2:**
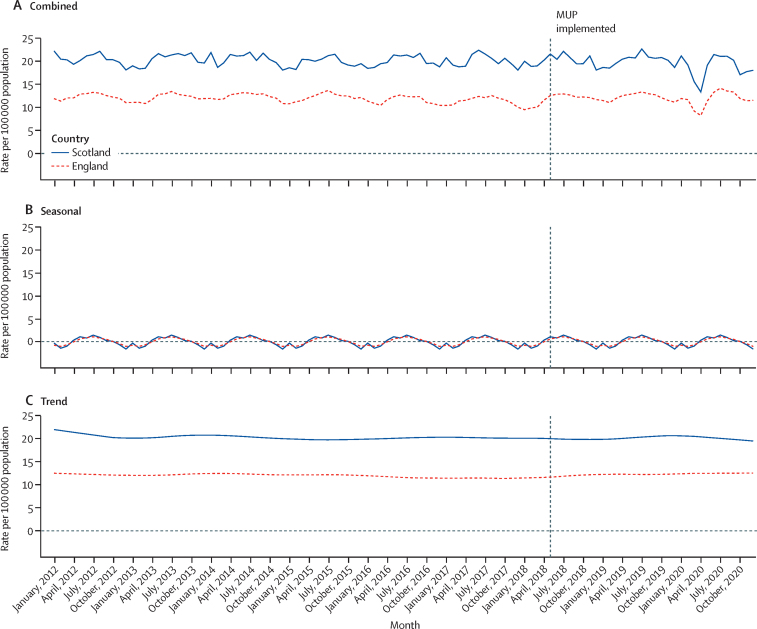
Rate of hospitalisations wholly attributable to alcohol consumption per 100 000 population by country Monthly rate (A), and decomposed seasonal (B) and trend (C) components. MUP=minimum unit pricing.

The rate of hospitalisations wholly attributable to alcohol consumption remained stable for both Scotland and England, for the entirety of the study period (figure 2). The decomposed trend rate was around 20 hospitalisations per month per 100 000 population in Scotland, almost double the decomposed trend rate for England. The rate dropped to the lowest point in April, 2020, the first month in which a national lockdown had been implemented in both countries as a result of the COVID-19 pandemic. Seasonality in the rate of hospitalisations wholly attributable to alcohol consumption was clear in Scotland and England, with rates peaking in July of each year. The contribution of seasonality to the overall time series was stable throughout the full study period.

In the 32 months following the implementation of MUP legislation, the policy was associated with a significant 13·4% decrease (95% CI –18·4 to –8·3; p=0·0004) in deaths wholly attributable to alcohol consumption compared with what would have been observed in the absence of MUP legislation (table 1). On average, 156 (–243 to –69) deaths wholly attributable to alcohol consumption were estimated to have been averted each year due to the implementation of MUP. These effects were driven by a significant decrease in chronic deaths wholly attributable to alcohol consumption (–14·9%, –20·8 to –8·5; p<0·0001). MUP implementation was also associated with significant reductions in deaths due to alcoholic liver disease (–11·7%, –16·7 to –6·4; p<0·0001) and alcohol dependence syndrome (–23·0%, –36·9 to –6·0; p=0·0093).

**Table 1:** Change in primary outcomes from controlled models associated with the implementation of alcohol minimum unit pricing legislation

		**Effect estimate, % (95% CI)**	**Effect estimate, N per year (95% CI)**	**p value**
**Deaths**				
All deaths		−13·4% (−18·4 to −8·3)	−156 (−243 to −69)	0·0004
Deaths from chronic causes		−14·9% (−20·8 to −8·5)	−186 (−253 to −119)	<0·0001
	Alcoholic liver disease	−11·7% (−16·7 to −6·4)	Not estimated	<0·0001
	Alcohol dependence syndrome	−23·0% (−36·9 to −6·0)	Not estimated	0·0093
Deaths from acute causes		6·6% (−13·7 to 31·8)	10 (−3 to 23)	0·55
**Hospitalisations**				
	All hospitalisations	−4·1% (−8·3 to 0·3)	−411 (−908 to 86)	0·064
	Hospitalisations for chronic causes	−7·3% (−9·5 to −4·9)	−622 (−880 to −364)	<0·0001
	Alcoholic liver disease	−9·8% (−17·5 to −1·3)	Not estimated	0·023
	Alcohol dependence syndrome	7·2% (0·3 to 14·7)	Not estimated	0·039
	Alcohol psychoses	−7·2% (−12·9 to −1·1)	Not estimated	0·019
	Alcohol misuse	−2·1% (−13·2 to 10·5)	Not estimated	0·73
Hospitalisations for acute causes		9·9% (−1·1 to 22·0)	146 (−65 to 357)	0·076
	Acute intoxication	3·9% (−11·0 to 21·2)	Not estimated	0·63

On average, and after 32 months, the implementation of alcohol MUP legislation was associated with a 4·1% decrease (95% CI –8·3 to 0·3; p=0·064) in hospitalisations wholly attributable to alcohol consumption (table 1). Contributing to this estimate, significant reductions in hospitalisations due to chronic conditions (–7·3%, –9·5 to –4·9; p<0·0001) were slightly offset with potential increases in hospitalisations due to acute conditions (9·9%, –1·1 to 22·0; p=0·076). MUP implementation was also associated with significant reductions in hospitalisations for alcoholic liver disease (–9·8%, –17·5 to –1·3; p=0·023) and alcohol psychoses (–7·2%, –12·9 to –1·1; p=0·019), as well as significant increases in hospitalisations for alcohol dependence syndrome (7·2%, 0·3 to 14·7; p=0·039). Further results on uncontrolled country-specific models can be found in the appendix (pp 8–9).

Subgroup analyses showed that MUP legislation was associated with reductions in deaths wholly attributable to alcohol consumption in males; females; 35–64 year olds; those aged 65 years and older; and the four most socioeconomically deprived decile groups (table 2). For hospitalisations wholly attributable to alcohol consumption, subgroup analyses indicated that MUP legislation was associated with reductions in males; 35–64 year olds; and the four most socioeconomically deprived decile groups. Further subgroup results for controlled and country-specific uncontrolled models can be found in the appendix (pp 5–13).

**Table 2:** Change in outcomes from controlled models associated with the implementation of alcohol minimum unit pricing legislation, by subgroup

	**Deaths wholly attributable to alcohol consumption**	**Hospitalisations wholly attributable to alcohol consumption**
**Sex**		
Males	−14·8% (−18·7 to −10·7)	−6·2% (−10·0 to −2·3)
Females	−12·0% (−20·5 to −2·6)	3·1% (−2·8 to 9·3)
**Age group**		
16–34 years	Not estimated	3·0% (−6·2 to 13·3)
35–64 years	−10·0% (−14·7 to −5·0)	−4·8% (−9·4 to 0·2)
≥65 years	−26·7% (−35·6 to −16·5)	−2·8% (−9·2 to 3·9)
**Deprivation decile**		
1 (Most deprived)	−21·6% (−31·8 to −10·0)	−6·8% (−11·9 to −1·3)
2	−17·5% (−27·5 to −5·9)	−4·5% (−10·8 to 2·3)
3	−33·6% (−43·4 to −22·1)	−6·3% (−11·3 to −1·0)
4	−19·3% (−29·4 to −7·7)	−6·9% (−11·4 to −2·3)
5	−9·7% (−27·2 to 12·2)	11·9% (−0·5 to 25·7)
6	−6·3% (−28·7 to 23·1)	−0·7% (−9·8 to 9·2)
7	−2·8% (−23·2 to 23·2)	0·7% (−7·6 to 9·7)
8	−9·2% (−28·3 to 14·8)	−1·2% (−8·1 to 6·4)
9	−2·9% (−23·5 to 23·2)	0·3% (−8·3 to 9·7)
10 (Least deprived)	−8·2% (−22·1 to 8·1)	−2·0% (−16·8 to 15·5)

Data are effect estimates, % (95% CI).

The effect size of the primary deaths outcome varied from –13·8% to –11·6% across sensitivity analyses (table 3). The effect size reduced (–2·1%, 95% CI –11·7 to 8·5) when we modelled the introduction of alcohol MUP legislation 6 months before the actual implementation, increasing confidence that the significant reduction in the primary deaths outcome was attributable to the implementation of alcohol MUP legislation.

**Table 3:** Change in outcomes from controlled models associated with the implementation of alcohol minimum unit pricing legislation, by sensitivity analyses

	**Deaths wholly attributable to alcohol consumption**	**Hospitalisations wholly attributable to alcohol consumption**
**Changes to model**		
Modelled using unobserved components model	−13·0% (−20·7 to −4·5)	−4·4% (−9·8 to 1·4)
Modelled difference between Scottish and English time series	−11·6% (−17·1 to −5·8)	−6·0% (−10·6 to −1·2)
Modelled pre-pandemic time series	−13·1% (−18·9 to −6·8)	−3·2% (−7·3 to 0·9)
**Changes to control group**		
North East England control group	−13·8% (−19·4 to −7·8)	−2·8% (−7·7 to 2·4)
North West England control group	−13·8% (−19·4 to −7·7)	0·5% (−5·2 to 6·5)
Non-geographical control group	−13·3% (−17·8 to −8·5)	2·9% (−0·4 to 6·3)
**Falsification test**		
Modelled implementation date 6 months earlier	−2·1% (−11·7 to 8·5)	−0·2% (−4·1 to 3·9)

Data are effect estimates, % (95% CI).

There was some variation in effect size of our primary hospitalisations outcome across sensitivity analyses (table 3). Using a different analytical method, and restricting the time series to pre-pandemic periods, yielded the most similar results. Furthermore, the effect size reduced (–0·2%, 95% CI –4·1 to 3·9) when we modelled the introduction of alcohol MUP legislation 6 months before the actual implementation, increasing confidence that the reduction in the primary hospitalisations outcome was attributable to the implementation of alcohol MUP legislation.

## Discussion

Across 32 months of implementation, we found a significant 13% reduction in deaths wholly attributable to alcohol consumption compared with our best estimate of what would have been expected had the legislation not been implemented. This is equivalent to avoiding 156 deaths per year, on average. There was a corresponding estimated reduction of 4% in hospitalisations for conditions wholly attributable to alcohol consumption, equivalent to avoiding 411 hospitalisations per year, on average. The use of a controlled interrupted time series study design allowed us to infer that the estimated impacts were plausible causal effects attributable to MUP legislation.

Exploratory analyses indicated that the largest reductions were estimated in the 40% most socioeconomically deprived areas in Scotland, indicating that the implementation of MUP has had a positive impact in tackling deprivation-based health inequalities in alcohol health harms. The implementation of MUP legislation was associated with reductions in deaths wholly attributable to alcohol consumption for males and females. Furthermore, we found associated reductions in the age groups of 35–64 years and 65 years and older, but were unable to evaluate change in the 16–34 years age group due to the relatively small number of deaths for this group. The positive impact of MUP legislation by population subgroup was generally similar for hospitalisations, although to a lesser degree.

We found potential indications that MUP was associated with a worsening of acute outcomes for deaths and hospitalisations wholly attributable to alcohol consumption. These findings are in contrast to findings from previous observational studies.^12^ Acute outcomes are a relatively small proportion of alcohol harms, around 5% of alcohol-specific deaths in Scotland, and these estimates therefore had a large degree of associated uncertainty.^3^ However, the findings were consistent across almost all subgroups. One identified plausible mechanism was that some subgroups reduced their spending on food or lowered their food intake due to the financial pressures of the policy being implemented, which might have led to faster intoxication or poisoning.^39^ Findings from another study offer another potential explanation, reporting evidence of switching of consumption from lower to higher alcohol-by-volume products (eg, cider to spirits), which could lead to quicker intoxication.^18^ These findings underscore the importance of ensuring timely, accessible services for those dependent on alcohol to coincide with the implementation of population-level policies. We estimated that reductions in chronic outcomes, particularly alcoholic liver disease, drove changes in total outcomes, offsetting the potential adverse consequence on acute outcomes. Taking both of these findings together indicates that the implementation of MUP has had a net benefit in reducing deaths and hospitalisations wholly attributable to alcohol consumption.

Our study has several strengths. Our study outcome definitions are wholly attributable to harmful levels of consumption of alcohol, and have been coded using internationally agreed definitions. We used high-quality data sources, pertaining to individual deaths and hospitalisations with recorded person-specific demographic attributes. Both UK countries under study have a universal health-care system free at the point of use, so there was minimal risk of sampling or recruitment bias. Many other health outcomes are known to be partially attributable to alcohol consumption, such as liver cancer and liver cirrhosis. The attributable fraction for these outcomes is estimated under the hypothetical assumption that the attributable fraction is based on reducing alcohol consumption to a theoretical minimum risk exposure level. Lag periods between changes in consumption and the occurrence of the attributable harm would vary across outcomes; for example, changes in liver cancer outcomes would take longer to emerge, relative to liver cirrhosis outcomes. Furthermore, alcohol consumption is only one of a range of possible causative factors that could influence the occurrence of these outcomes. In the context of our study design with monthly outcomes, there would be increased uncertainty over associating changes in partially attributable outcomes with the implementation of MUP. In terms of other possible study outcomes, all-cause mortality is a frequently used outcome. However, it has been suggested that evaluating changes in all-cause mortality due to changes in alcohol consumption might not be suitable, given that the distribution of all-cause mortality in those at risk of alcohol health harms is different to the distribution in society as a whole.^40^

Study outcomes were assessed using a controlled interrupted time series study design, allowing us to determine the difference between outcomes we observed and our best representation of what might have happened under the counterfactual situation that MUP legislation was not enacted in Scotland. We therefore have increased certainty that our findings were associated with the implementation of MUP legislation, rather than unexplained factors impacting the occurrence of alcohol health harms. There were no biases related to the timing of, or deviation from, the policy intervention or measurement of study outcomes.^41^ Bias due to confounding was minimised through measuring outcomes over a long pre-intervention study period in monthly increments, to appropriately characterise pre-intervention trends and patterns, in both countries. As the MUP policy is highly publicised, the context and awareness regarding the policy was not experienced equally in Scotland and England. However, this is unlikely to have influenced the recording of study outcomes, as records for deaths and hospital diagnoses are coded using agreed standards. Our choice of control group was England, a neighbouring country with the same UK government, economy, and culture. We incorporated the level of country-specific COVID-19-related restrictions, which meant that the timing of restrictions was well aligned with England as a whole. Using regions of England as our primary control group would have potentially meant that differences in timing of regional COVID-19 waves, and subsequent pressures on services, might not have always aligned well with country-level restrictions. However, we also did sensitivity analyses by altering our control group to the North East and North West regions of England, areas that directly border Scotland. Although cross-border purchases from Scotland to England might have occurred, they are likely to be infrequent, and unlikely to have a major impact on our reported effect sizes due to the low population density around the Scottish–English border.^42^, ^43^

Part of our time series data covered a period of the COVID-19 pandemic. During this period, restrictions on the purchasing of alcohol were imposed for on-trade premises (eg, pubs and restaurants).^44^ Although on-trade premises were largely unaffected by MUP, these restrictions had the potential to affect the level of alcohol health harms. Furthermore, COVID-19 and associated protection measures had a substantial impact on hospital capacity, and individuals reported increased reluctance in interacting with health-care services for immediate medical concerns during this period.^45^, ^46^ We adjusted for temporal and country-specific differences in the extent of restrictions, and acknowledge that the COVID-19 pandemic increases the uncertainty of our findings related to hospitalisations. We included sensitivity analyses to assess the impact of MUP legislation before the COVID-19 pandemic, as a means of removing the pandemic-related uncertainty, which yielded similar effect sizes as our main analyses. We also did a range of pre-specified sensitivity analyses to test the robustness of the study findings and obtained largely similar results, validating the interpretation of the main study findings. However, we found some variations across sensitivity analyses for the primary hospitalisations outcome relating to different geographical choices of a control group. This might, in part, be explained by differences in the timing of regional waves of COVID-19 cases leading to temporal differences in pressures on hospital capacity.

A previous study has indicated that the implementation of MUP legislation reduced alcohol sales by 3%.^21^ This finding relates to the change in overall population-level alcohol sales, and might mask important subgroup-specific changes in sales and consumption. Indeed, the greatest reductions in alcohol purchasing were observed in households that purchased the most alcohol before the policy was implemented, with very little or no impact on those purchasing at lower levels.^17^, ^42^, ^47^ This is consistent with the theory of change that underpinned the legislation. Our study reports on the final intended outcome and finds that this reduction in sales led to a 13% reduction in deaths and a 4% reduction in hospitalisations. The methods used suggest plausibility that these effects can be causally attributed to MUP.^48^ Our findings are most positive for socioeconomically deprived groups and males, which is consistent with the intended impact of MUP legislation in tackling subgroups experiencing disproportionately high levels of alcohol health harms. Previous evidence has illustrated that the subgroups experiencing the greatest alcohol health harms tend to purchase the cheapest alcohol.^11^ Furthermore, most dose–response curves between consumption and alcohol health harms are exponential, meaning that risk reductions are greater for changes in heavier drinkers, compared with moderate drinkers, if both groups were to reduce their consumption by the same level.^49^ Our findings show larger relative effect sizes for deaths than hospitalisations outcomes. This could partly be because increasing the lifespan of individuals by averting alcohol-attributable deaths might increase the need for health-care support. It is likely that the deaths that were averted were in subgroups that remain vulnerable to alcohol health harms and require additional preventive, planned, and unplanned support from health-care services.

Published estimates have indicated a recent worsening in alcohol-specific mortality in both Scotland and England.^2^ Our study period did not include these recent data. However, the increase in the rate in Scotland from 2020 to 2021 (4%) was lower than in England (7%). It is therefore unlikely that the inclusion of more recent data would have altered our main findings.

In conclusion, the implementation of MUP legislation in Scotland has led to significant overall reductions in deaths, and reductions in hospitalisations, wholly attributable to alcohol consumption. Furthermore, the legislation has had a positive impact in tackling alcohol-related health inequalities.

## Data sharing

Metadata relating to the source data used in this manuscript have been previously published by the data controllers (Scottish deaths: https://www.nrscotland.gov.uk/statistics-and-data/statistics/statistics-by-theme/vital-events/deaths/deaths-background-information; English deaths: https://www.ons.gov.uk/peoplepopulationandcommunity/birthsdeathsandmarriages/deaths/methodologies/userguidetomortalitystatisticsjuly2017; Scottish hospitalisations: https://www.ndc.scot.nhs.uk/Data-Dictionary/SMR-Datasets; English hospitalisations: https://digital.nhs.uk/data-and-information/data-tools-and-services/data-services/hospital-episode-statistics). Requests to access the data used in this study can be made through formal application to the relevant agencies (Scottish data: Public Health Scotland; English hospitalisations: NHS Digital; English deaths: Office for National Statistics Secure Research Service). Further results relating to pre-specified partially attributable outcomes will be reported by Public Health Scotland on March 21, 2023.

## Declaration of interests

GMAW, CF, CB, and LG report funding from the Scottish Government. All other authors declare no competing interests.
