# Supplementary material for: Evaluating the impact of alcohol minimum unit pricing on deaths and hospitalisations in Scotland: a controlled interrupted time series study
Source: Lancet. 2023 Apr 22;401(10385):1361–70. doi: 10.1016/S0140-6736(23)00497-X (PMC10154457; doi:10.1016/S0140-6736(23)00497-X)
Supplement: Supplementary appendix [file mmc1.pdf]

THE LANCET

Supplementary appendix

This appendix formed part of the original submission and has been peer reviewed. We post it as supplied by the authors.

Supplement to: Wyper GMA, Mackay DF, Fraser C, et al. Evaluating the impact of alcohol minimum unit pricing on deaths and hospitalisations in Scotland: a controlled interrupted time series study. *Lancet* 2023; published online March 21. https://doi.org/10.1016/S0140-6736(23)00497-X.

Supplementary material

This appendix has been provided by the authors to give readers additional information about their work.

Supplement to:

Wyper GMA, Mackay DF, Fraser C, Lewsey J, Robinson M, Beeston C, Giles L. Evaluating the impact of alcohol minimum unit pricing on deaths and hospitalisations in Scotland: a controlled interrupted time series study. *Lancet* 2023; published online March 21. https://doi.org/10.1016/S0140-6736(23)00497-X.

Contents

Contents	Page
**Figure S1**. Study flow diagram	3
**Table S1**. ICD-10 code definitions of study outcomes	4
**Table S2**. Change in outcomes from controlled models associated with the implementation of alcohol minimum unit pricing legislation, by sex	5
**Table S3**. Change in outcomes from controlled models associated with the implementation of alcohol minimum unit pricing legislation, by age-group	6
**Table S4**. Change in death outcomes from controlled models associated with the implementation of alcohol minimum unit pricing legislation, by socio-economic deprivation decile	7
**Table S5**. Change in hospitalisation outcomes from controlled models associated with the implementation of alcohol minimum unit pricing legislation, by socio-economic deprivation decile	7
**Table S6**. Change in outcomes associated with alcohol minimum unit pricing legislation, by sex, Scotland uncontrolled model	8
**Table S7**. Change in outcomes associated with alcohol minimum unit pricing legislation, by sex, England uncontrolled model	9
**Table S8**. Change in outcomes associated with alcohol minimum unit pricing legislation, by age-group, Scotland uncontrolled model	10
**Table S9**. Change in outcomes associated with alcohol minimum unit pricing legislation, by age-group, England uncontrolled model	11
**Table S10**. Change in death outcomes associated with alcohol minimum unit pricing legislation, by socio-economic deprivation decile, Scotland uncontrolled model	12
**Table S11**. Change in death outcomes associated with alcohol minimum unit pricing legislation, by socio-economic deprivation decile, England uncontrolled model	12
**Table S12**. Change in hospitalisation outcomes associated with alcohol minimum unit pricing legislation, by socio-economic deprivation decile, Scotland uncontrolled model	13
**Table S13**. Change in hospitalisation outcomes associated with alcohol minimum unit pricing legislation, by socio-economic deprivation decile, England uncontrolled model	13
**Data permissions and Statistical Analysis Plan**	14
**STROBE statement**	15

Figure S1. Study flow diagram

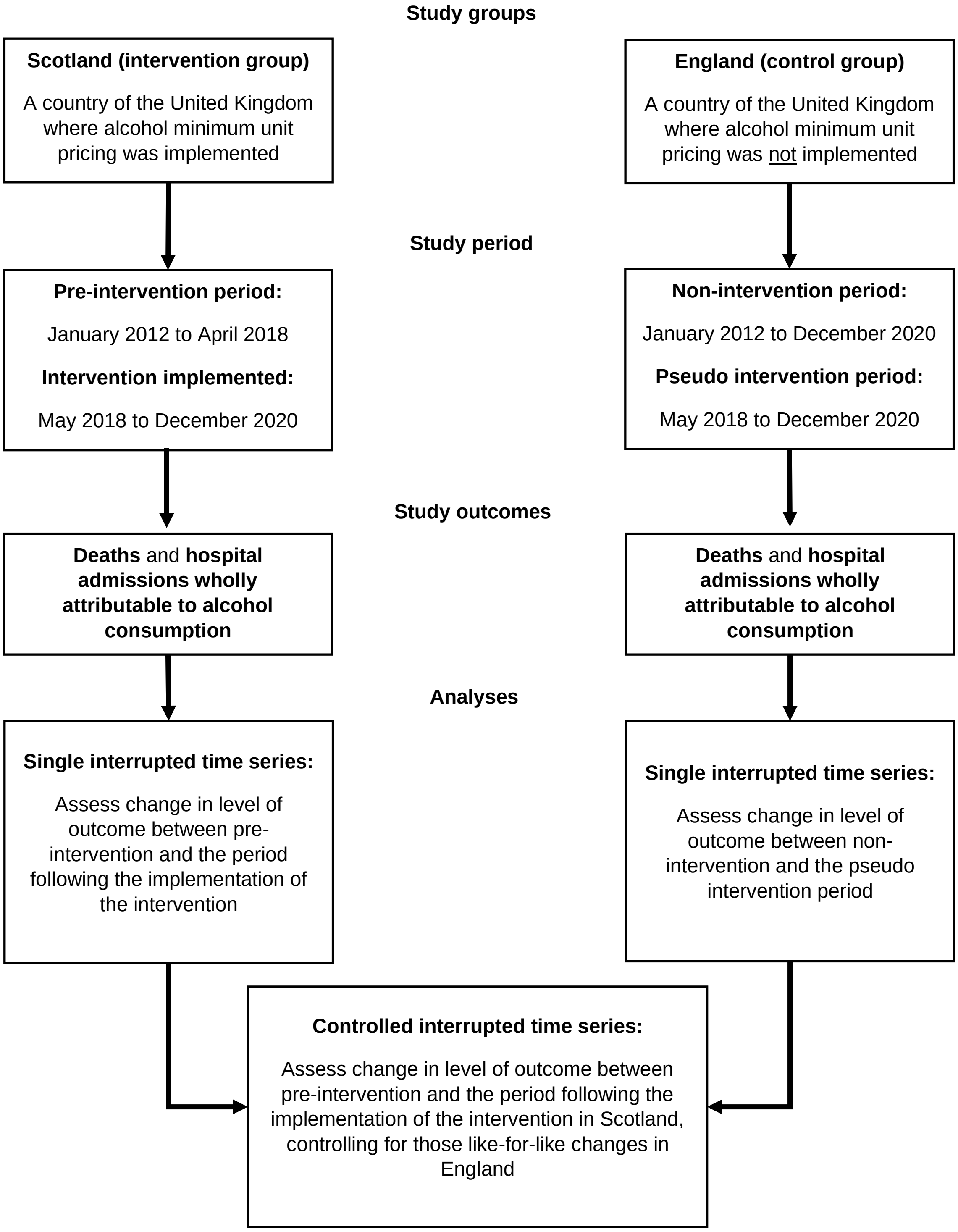

Table S1. ICD-10 code definitions of study outcomes

Type	Study outcome	ICD-10 definition
**All outcomes wholly attributable to alcohol consumption**	**Primary outcome**	**E24·4, F10·1, F10·2, F10·3-F10·9, G31·2, G62·1, G72·1, I42·6, K29·2, K70, K85·2, K86·0, F10·0, T51·0, T51·1, T51·2, T51·3, T51·8, T51·9, X45, Y15, X65, R78·0, Y90**
Chronic outcomes wholly attributable to alcohol consumption	All chronic outcomes	E24·4, F10·1, F10·2, F10·3-F10·9, G31·2, G62·1, G72·1, I42·6, K29·2, K70, K85·2, K86·0
	Alcoholic liver disease	K70
	Alcohol dependence syndrome	F10·2
	Alcohol psychoses	F10·3-F10·9
	Alcohol misuse	F10·1
Acute outcomes wholly attributable to alcohol consumption	All acute outcomes	F10·0, T51·0, T51·1, T51·2, T51·3, T51·8, T51·9, X45, Y15, X65, R78·0, Y90
	Acute intoxication	F10·0

Table S2. Change in outcomes from controlled models associated with the implementation of alcohol minimum unit pricing legislation, by sex

**Study outcome**	**Effect estimate, % (95% confidence interval)**		
	**Both sexes**	**Males**	**Females**
**All deaths**	**-13·4% (-18·4% to -8·3%)**	**-14·8% (-18·7% to -10·7%)**	**-12·0% (-20·5% to -2·6%)**
Deaths from chronic causes	-14·9% (-20·8% to -8·5%)	-18·1% (-23·5% to -12·8%)	-12·6% (-21·3% to -3·1%)
Alcoholic liver disease	-11·7% (-16·7% to -6·4%)	-15·6% (-21·3% to -9·5%)	-10·5% (-18·8% to -1·5%)
Alcohol dependence syndrome	-23·0% (-36·9% to -6·0%)	-19·9% (-39·5% to 6·1%)	-5·1% (-9·7% to -0·2%)
Alcohol psychoses	Not estimated	Not estimated	Not estimated
Alcohol misuse	Not estimated	Not estimated	Not estimated
Deaths from acute causes	6·6% (-13·7% to 31·8%)	4·4% (-1·5% to 10·6%)	0·2% (-3·5% to 4·2%)
Acute intoxication	Not estimated	Not estimated	Not estimated
**All hospitalisations**	**-4·1% (-8·3% to 0·3%)**	**-6·2% (-10·0% to -2·3%)**	**3·1% (-2·8% to 9·3%)**
Hospitalisations for chronic causes	-7·3% (-9·5% to -4·9%)	-9·7% (-11·9% to -7·5%)	0·0% (-6·1% to 6·4%)
Alcoholic liver disease	-9·8% (-17·5% to -1·3%)	-11·4% (-20·5% to -1·2%)	4·6% (-6·2% to 16·6%)
Alcohol dependence syndrome	7·2% (0·3% to 14·7%)	2·0% (-4·6% to 9·1%)	14·0% (3·8% to 25·1%)
Alcohol psychoses	-7·2% (-12·9% to -1·1%)	-8·4% (-13·2% to -3·2%)	-0·8% (-7·3% to 6·1%)
Alcohol misuse	-2·1% (-13·2% to 10·5%)	-0·9% (-12·9% to 12·6%)	0·0% (-12·9% to 14·8%)
Hospitalisations for acute causes	9·9% (-1·1% to 22·0%)	8·5% (-3·3% to 22·1%)	15·6% (2·1% to 30·9%)
Acute intoxication	3·9% (-11·0% to 21·2%)	7·7% (-5·4% to 22·4%)	12·2% (-0·6% to 26·7%)

Table S3. Change in outcomes from controlled models associated with the implementation of alcohol minimum unit pricing legislation, by age-group

Study outcome	Effect estimate, % (95% confidence interval)		
	16–34 years	35–64 years	≥65 years
**All deaths**	**Not estimated**	**-10·0% (-14·7% to -5·0%)**	**-26·7% (-35·6% to -16·5%)**
Deaths from chronic causes	Not estimated	-12·1% (-16·7% to -7·2%)	-27·7% (-36·9% to -17·2%)
Alcoholic liver disease	Not estimated	-4·9% (-9·7% to 0·2%)	-24·0% (-36·0% to -9·8%)
Alcohol dependence syndrome	Not estimated	-26·7% (-44·7% to -2·8%)	-15·3% (-25·2% to -4·1%)
Alcohol psychoses	Not estimated	Not estimated	-14·4% (-21·1% to -7·1%)
Alcohol misuse	Not estimated	Not estimated	Not estimated
Deaths from acute causes	Not estimated	4·8% (-2·3% to 12·6%)	Not estimated
Acute intoxication	Not estimated	Not estimated	Not estimated
**All hospitalisations**	**3·0% (-6·2% to 13·3%)**	**-4·8% (-9·4% to 0·2%)**	**-2·8% (-9·2% to 3·9%)**
Hospitalisations for chronic causes	-2·8% (-9·7% to 4·7%)	1·0% (-6·0% to 8·5%)	-4·7% (-10·1% to 1·4%)
Alcoholic liver disease	11·0% (-8·8% to 35·1%)	2·3% (-2·6% to 7·4%)	7·0% (-8·1% to 24·6%)
Alcohol dependence syndrome	2·4% (-11·8% to 18·9%)	6·4% (-1·2% to 14·6%)	0·7% (-19·7% to 26·4%)
Alcohol psychoses	-5·5% (-16·7% to 7·1%)	-7·6% (-12·7% to -2·2%)	-9·6% (-22·9% to 6·1%)
Alcohol misuse	6·6% (-14·8% to 33·5%)	-9·0% (-20·2% to 3·9%)	16·8% (-1·8% to 38·8%)
Hospitalisations for acute causes	13·0% (-0·8% to 28·5%)	12·9% (-1·2% to 25·7%)	9·6% (-4·6% to 26·1%)
Acute intoxication	12·2% (-4·0% to 31·1%	2·8% (-12·0% to 20·2%)	13·5% (-1·5% to 30·9%)

Table S4. Change in death outcomes from controlled models associated with the implementation of alcohol minimum unit pricing legislation, by socio-economic deprivation decile

Deprivation decile	Effect estimate, % (95% confidence interval)			
	All deaths	Deaths from chronic causes	Alcoholic liver disease	Deaths from acute causes
Decile 1 [Most deprived]	**-21·6% (-31·8% to -10·0%)**	-23·8% (-33·7% to -12·5%)	-16·3% (-30·9% to 1·2%)	Not estimated
Decile 2	**-17·5% (-27·5% to -5·9%)**	-16·2% (-32·2% to 3·3%)	-14·8% (-33·8% to 9·6%)	Not estimated
Decile 3	**-33·6% (-43·4% to -22·1%)**	-31·8% (-41·7% to -20·2%)	-37·2% (-48·9% to -22·9%)	Not estimated
Decile 4	**-19·3% (-29·4% to -7·7%)**	-17·7% (-32·4% to 0·1%)	2·4% (-13·9% to 21·7%)	Not estimated
Decile 5	**-9·7% (-27·2% to 12·2%)**	-10·8% (-23·7% to 4·3%)	-15·0% (-32·7% to 7·1%)	Not estimated
Decile 6	**-6·3% (-28·7% to 23·1%)**	-2·4% (-5·7% to 1·2%)	14·1% (-11·6% to 47·6%)	Not estimated
Decile 7	**-2·8% (-23·2% to 23·2%)**	-4·3% (-25·4% to 22·7%)	-2·8% (-21·6% to 20·5%)	Not estimated
Decile 8	**-9·2% (-28·3% to 14·8%)**	-12·1% (-29·0% to 10·0%)	-12·1% (-30·4% to 10·9%)	Not estimated
Decile 9	**-2·9% (-23·5% to 23·2%)**	-4·5% (-20·5% to 14·3%)	-3·3% (-20·1% to 17·0%)	Not estimated
Decile 10 [Least deprived]	**-8·2% (-22·1% to 8·1%)**	-10·6% (-27·3% to 9·9%)	-5·9% (-21·4% to 12·7%)	Not estimated

Table S5. Change in hospitalisation outcomes from controlled models associated with the implementation of alcohol minimum unit pricing legislation, by socio-economic deprivation decile

Deprivation decile	Effect estimate, % (95% confidence interval)			
	All hospitalisations	Hospitalisations for chronic causes	Alcoholic liver disease	Hospitalisations for acute causes
Decile 1 [Most deprived]	**-6·8% (-11·9% to -1·3%)**	-2·7% (-8·5% to 3·6%)	-9·9% (-19·1% to 0·3%)	7·1% (-6·3% to 22·5%)
Decile 2	**-4·5% (-10·8% to 2·3%)**	-8·4% (-13·4% to -3·2%)	-3·7% (-16·2% to 10·7%)	9·9% (-4·5% to 26·5%)
Decile 3	**-6·3% (-11·3% to -1·0%)**	-9·2% (-15·1% to -2·8%)	-28·3% (-37·0% to -18·4%)	24·0% (5·8% to 45·5%)
Decile 4	**-6·9% (-11·4% to -2·3%)**	-13·2% (-20·7% to -5·0%)	-34·4% (-46·0% to -20·5%)	34·3% (13·7% to 58·6%)
Decile 5	**11·9% (-0·5% to 25·7%)**	10·2% (-2·4% to 24·5%)	8·1% (-10·7% to 31·0%)	16·0% (2·8% to 30·9%)
Decile 6	**-0·7% (-9·8% to 9·2%)**	0·9% (-1·8% to 3·7%)	2·6% (-12·8% to 20·8%)	0·5% (-24·4% to 33·6%)
Decile 7	**0·7% (-7·6% to 9·7%)**	3·4% (-6·5% to 14·1%)	-4·8% (-17·5% to 9·7%)	9·7% (-13·2% to 38·7%)
Decile 8	**-1·2% (-8·1% to 6·4%)**	-3·5% (-10·9% to 4·4%)	2·2% (-29·8% to 48·9%)	43·5% (9·6% to 87·8%)
Decile 9	**0·3% (-8·3% to 9·7%)**	0·4% (-0·1% to 0·9%)	-14·1% (-28·3% to 2·8%)	3·0% (-1·2% to 7·6%)
Decile 10 [Least deprived]	**-2·0% (-16·8% to 15·5%)**	-3·8% (-12·0% to 5·1%)	29·4% (8·6% to 54·2%)	7·7% (-15·5% to 37·4%)

Table S6. Change in outcomes associated with alcohol minimum unit pricing legislation, by sex, Scotland uncontrolled model

Study outcome	Effect estimate, % (95% confidence interval)		
	Both sexes	Males	Females
**All deaths**	**-14·0% (-18·4% to -9·3%)**	**-18·1% (-22·5% to -13·5%)**	**-11·0% (-19·7% to -1·3%)**
Deaths from chronic causes	-16·3% (-21·7% to -10·4%)	-20·3% (-25·0% to -15·4%)	-13·2% (-21·9% to -3·4%)
Alcoholic liver disease	-15·0% (-18·8% to -11·0%)	-17·4% (-27·5% to -5·9%)	-11·8% (-18·0% to -5·1%)
Alcohol dependence syndrome	-26·0% (-40·1% to -8·6%)	-28·0% (-44·2% to -7·2%)	-4·8% (-9·4% to 0·0%)
Alcohol psychoses	Not estimated	Not estimated	Not estimated
Alcohol misuse	Not estimated	Not estimated	Not estimated
Deaths from acute causes	6·3% (-21·8% to 44·5%)	4·6% (-1·3% to 10·9%)	0·4% (-3·2% to 4·1%)
Acute intoxication	Not estimated	Not estimated	Not estimated
**All hospitalisations**	**2·5% (-0·5% to 5·5%)**	**-0·7% (-2·9% to 1·6%)**	**7·3% (2·5% to 12·4%)**
Hospitalisations for chronic causes	-0·9% (-3·8% to 2·0%)	-3·3% (-6·7% to 0·2%)	4·3% (-6·3% to 16·1%)
Alcoholic liver disease	-7·4% (-15·0% to 0·9%)	-15·1% (-25·9% to -2·7%)	15·5% (5·9% to 25·9%)
Alcohol dependence syndrome	9·9% (2·4% to 17·9%)	7·0% (-2·1% to 16·8%)	4·6% (-3·9% to 13·8%)
Alcohol psychoses	-3·7% (-13·7% to 7·5%)	-4·1% (-11·3% to 3·6%)	-4·8% (-17·2% to 9·5%)
Alcohol misuse	-0·2% (-11·1% to 12·0%)	5·7% (-5·1% to 17·8%)	26·4% (10·4% to 44·1%)
Hospitalisations for acute causes	18·1% (7·3% to 30·0%)	21·4% (10·4% to 33·5%)	21·9% (9·9% to 35·3%)
Acute intoxication	17·0% (4·4% to 31·2%)	20·9% (9·7% to 33·2%)	-11·0% (-19·7% to -1·3%)

Table S7. Change in outcomes associated with alcohol minimum unit pricing legislation, by sex, England uncontrolled model

**Study outcome**	**Effect estimate, % (95% confidence interval)**		
	**Both sexes**	**Males**	**Females**
**All deaths**	**-5·7% (-9·4% to -1·8%)**	**-5·0% (-9·8% to 0·2%)**	**-7·5% (-13·1% to -2·0%)**
Deaths from chronic causes	-6·0% (-10·0% to -2·0%)	-5·0% (-9·0% to -0·8%)	-10·4% (-16·1% to -4·5%)
Alcoholic liver disease	-6·4% (-10·1% to -2·6%)	-5·7% (-9·4% to -1·9%)	-8·0% (-13·3% to -2·3%)
Alcohol dependence syndrome	1·4% (-7·8% to 11·4%)	-7·3% (-21·5% to 9·4%)	7·5% (-16·1% to 37·7%)
Alcohol psychoses	Not estimated	Not estimated	Not estimated
Alcohol misuse	Not estimated	Not estimated	Not estimated
Deaths from acute causes	16·4% (0·2% to 35·1%)	2·7% (-15·6% to 25·5%)	33·5% (7·1% to 66·5%)
Acute intoxication	Not estimated	Not estimated	Not estimated
**All hospitalisations**	**8·4% (2·9% to 14·2%)**	**13·3% (-1·6% to 30·5%)**	**7·1% (0·8% to 13·9%)**
Hospitalisations for chronic causes	7·8% (4·7% to 11·1%)	7·8% (4·4% to 11·2%)	8·4% (4·3% to 12·6%)
Alcoholic liver disease	7·8% (-23·0% to 50·8%)	7·5% (1·3% to 14·0%)	-0·4% (-3·9% to 3·3%)
Alcohol dependence syndrome	13·4% (-12·5% to 46·8%)	17·1% (-4·0% to 42·9%)	4·8% (-13·4% to 26·7%)
Alcohol psychoses	10·0% (4·0% to 16·2%)	8·2% (0·6% to 16·4%)	13·0% (6·9% to 19·4%)
Alcohol misuse	15·4% (6·2% to 25·4%)	26·7% (17·9% to 36·1%)	8·3% (-2·3% to 20·1%)
Hospitalisations for acute causes	9·6% (0·9% to 19·1%)	7·1% (-6·7% to 23·1%)	4·1% (-10·1% to 20·4%)
Acute intoxication	5·2% (-7·7% to 19·8%)	12·9% (-4·1% to 32·8%)	13·7% (-2·1% to 31·9%)

Table S8. Change in outcomes associated with alcohol minimum unit pricing legislation, by age-group, Scotland uncontrolled model

**Study outcome**	**Effect estimate, % (95% confidence interval)**		
	**16–34 years**	**35–64 years**	**≥65 years**
**All deaths**	**Not estimated**	**-8·0% (-11·7% to -4·1%)**	**-33·8% (-39·9% to -27·2%)**
Deaths from chronic causes	Not estimated	-12·7% (-16·5% to -8·7%)	-34·8% (-41·4% to -27·5%)
Alcoholic liver disease	Not estimated	-11·0% (-15·9% to -5·9%)	-24·7% (-36·6% to -10·6%)
Alcohol dependence syndrome	Not estimated	-27·8% (-48·5% to 1·1%)	-17·1% (-29·8% to -1·2%)
Alcohol psychoses	Not estimated	Not estimated	-15·0% (-21·2% to -8·4%)
Alcohol misuse	Not estimated	Not estimated	Not estimated
Deaths from acute causes	Not estimated	4·8% (-1·3% to 11·4%)	Not estimated
Acute intoxication	Not estimated	Not estimated	Not estimated
**All hospitalisations**	**12·8% (4·1% to 22·2%)**	**0·6% (-1·9% to 3·2%)**	**0·4% (-6·6% to 8·0%)**
Hospitalisations for chronic causes	8·2% (3·8% to 12·9%)	-2·5% (-5·7% to 0·8%)	-4·3% (-10·9% to 3·1%)
Alcoholic liver disease	14·5% (-5·4% to 38·5%)	-11·1% (-19·3% to -2·0%)	-3·1% (-16·4% to 12·4%)
Alcohol dependence syndrome	12·7% (-0·6% to 27·7%)	9·4% (2·7% to 16·6%)	3·4% (-17·7% to 29·8%)
Alcohol psychoses	0·8% (0·0% to 1·6%)	-3·9% (-12·4% to 5·6%)	-8·7% (-23·5% to 9·0%)
Alcohol misuse	8·8% (-10·6% to 32·4%)	-3·6% (-15·6% to 10·1%)	-9·5% (-25·5% to 10·0%)
Hospitalisations for acute causes	22·1% (9·1% to 36·9%)	12·8% (-6·8% to 36·6%)	20·9% (-1·7% to 48·7%)
Acute intoxication	23·2% (2·5% to 48·0%)	15·1% (-1·0% to 33·8%)	20·8% (5·5% to 38·3%)

Table S9. Change in outcomes associated with alcohol minimum unit pricing legislation, by age-group, England uncontrolled model

Study outcome	Effect estimate, % (95% confidence interval)		
	16–34 years	35–64 years	≥65 years
**All deaths**	**Not estimated**	**-0·3% (-4·6% to 4·1%)**	**-9·8% (-16·3% to -2·8%)**
Deaths from chronic causes	Not estimated	-2·0% (-5·9% to 2·1%)	-9·4% (-18·8% to 1·1%)
Alcoholic liver disease	Not estimated	-3·5% (-7·5% to 0·7%)	-10·9% (-20·2% to -0·4%)
Alcohol dependence syndrome	Not estimated	3·0% (-9·7% to 17·5%)	-0·3% (-1·3% to 0·7%)
Alcohol psychoses	Not estimated	Not estimated	0·3% (-1·2% to 1·7%)
Alcohol misuse	Not estimated	Not estimated	Not estimated
Deaths from acute causes	Not estimated	2·1% (-0·7% to 4·9%)	Not estimated
Acute intoxication	Not estimated	Not estimated	Not estimated
**All hospitalisations**	**17·0% (10·6% to 23·9%)**	**8·7% (3·5% to 14·1%)**	**7·7% (-0·2% to 15·6%)**
Hospitalisations for chronic causes	23·7% (14·3% to 33·8%)	8·2% (3·8% to 12·7%)	5·0% (-1·3% to 11·7%)
Alcoholic liver disease	3·4% (-7·7% to 15·3%)	3·8% (-1·6% to 9·5%)	4·6% (-5·2% to 15·4%)
Alcohol dependence syndrome	24·6% (9·2% to 42·9%)	15·0% (-2·6% to 35·9%)	5·8% (-9·8% to 24·2%)
Alcohol psychoses	15·8% (7·8% to 24·6%)	10·4% (4·0% to 17·2%)	2·7% (-3·6% to 9·4%)
Alcohol misuse	30·2% (17·8% to 43·8%)	15·5% (7·3% to 24·4%)	6·1% (-5·4% to 18·9%)
Hospitalisations for acute causes	9·9% (-1·8% to 22·8%)	5·7% (-7·0% to 20·2%)	18·3% (2·3% to 37·0%)
Acute intoxication	13·5% (-2·6% to 32·2%)	3·1% (-11·5% to 20·2%)	23·1% (3·7% to 46·2%)

Table S10. Change in death outcomes associated with alcohol minimum unit pricing legislation, by socio-economic deprivation decile, Scotland uncontrolled model

Deprivation decile	Effect estimate, % (95% confidence interval)			
	All deaths	Deaths from chronic causes	Alcoholic liver disease	Deaths from acute causes
Decile 1 [Most deprived]	**-17·8% (-29·1% to -4·7%)**	-20·2% (-30·7% to -8·1%)	-18·7% (-32·7% to -1·9%)	Not estimated
Decile 2	**-18·3% (-28·8% to -6·2%)**	-16·2% (-28·5% to -1·9%)	-15·1% (-33·6% to 8·7%)	Not estimated
Decile 3	**-36·4% (-46·5% to -24·4%)**	-33·0% (-42·4% to -21·9%)	-35·6% (-47·3% to -21·2%)	Not estimated
Decile 4	**-19·5% (-28·5% to -9·4%)**	-20·2% (-31·0% to -7·7%)	-3·2% (-18·9% to 15·6%)	Not estimated
Decile 5	**-8·1% (-25·6% to 13·5%)**	-10·1% (-20·5% to 1·5%)	-8·2% (-27·7% to 16·5%)	Not estimated
Decile 6	**-7·6% (-29·6% to 21·2%)**	4·4% (-19·1% to 34·7%)	9·4% (-15·3% to 41·5%)	Not estimated
Decile 7	**-3·8% (-22·7% to 19·6%)**	-5·1% (-25·5% to 20·9%)	-2·4% (-22·8% to 23·4%)	Not estimated
Decile 8	**-8·3% (-27·2% to 15·5%)**	-9·7% (-27·1% to 46·9%)	-5·4% (-18·5% to 9·8%)	Not estimated
Decile 9	**-2·8% (-23·4% to 23·3%)**	-7·8% (-27·6% to 17·4%)	-3·5% (-20·2% to 16·7%)	Not estimated
Decile 10 [Least deprived]	**-8·4% (-21·6% to 7·0%)**	-11·0% (-27·7% to -9·6%)	-4·5% (-23·6% to 19·2%)	Not estimated

Table S11. Change in death outcomes associated with alcohol minimum unit pricing legislation, by socio-economic deprivation decile, England uncontrolled model

Deprivation decile	Effect estimate, % (95% confidence interval)			
	All deaths	Deaths from chronic causes	Alcoholic liver disease	Deaths from acute causes
Decile 1 [Most deprived]	**-8·8% (-16·0% to -1·0%)**	-10·8% (-17·4% to -3·6%)	-12·7% (-18·9% to -6·0%)	Not estimated
Decile 2	**-4·9% (-12·1% to 2·9%)**	-6·1% (-12·5% to 0·8%)	-6·8% (-13·2% to 0·2%)	Not estimated
Decile 3	**-0·8% (-9·2% to 8·3%)**	-4·8% (-13·5% to 4·9%)	0·9% (-9·2% to 12·1%)	Not estimated
Decile 4	**-4·8% (-10·7% to 1·6%)**	-4·2% (-10·6% to 2·6%)	-2·8% (-9·6% to 4·4%)	Not estimated
Decile 5	**6·3% (-0·8% to 14·0%)**	6·4% (-2·4% to 15·8%)	4·5% (-4·6% to 14·5%)	Not estimated
Decile 6	**-3·7% (-9·8% to 2·7%)**	-11·6% (-21·7% to 0·0%)	-8·1% (-18·5% to 3·7%)	Not estimated
Decile 7	**-0·4% (-6·1% to 5·5%)**	-1·1% (-6·9% to 5·0%)	-1·7% (-6·9% to 3·8%)	Not estimated
Decile 8	**-4·3% (-8·4% to -0·1%)**	-4·7% (-7·8% to -1·6%)	-3·3% (-6·1% to -0·4%)	Not estimated
Decile 9	**0·7% (-5·0% to 6·8%)**	-0·9% (-6·7% to 5·2%)	-2·7% (-9·6% to 4·7%)	Not estimated
Decile 10 [Least deprived]	**-0·5% (-4·2% to 3·3%)**	-3·1% (-6·2% to 0·2%)	-0·2% (-4·6% to 4·4%)	Not estimated

Table S12. Change in hospitalisation outcomes associated with alcohol minimum unit pricing legislation, by socio-economic deprivation decile, Scotland uncontrolled model

Deprivation decile	Effect estimate, % (95% confidence interval)			
	**All hospitalisations**	**Hospitalisations for chronic causes**	**Alcoholic liver disease**	**Hospitalisations for acute causes**
Decile 1 [Most deprived]	**-0·6% (-5·4% to 4·5%)**	-1·0% (-6·9% to 5·3%)	-9·6% (-19·4% to 1·3%)	7·5% (-4·4% to 20·9%)
Decile 2	**-0·3% (-7·2% to 7·1%)**	-5·7% (-8·2% to -3·2%)	-10·9% (-23·2% to 3·4%)	15·7% (4·1% to 28·5%)
Decile 3	**0·3% (-9·8% to 11·6%)**	-4·6% (-10·5% to 1·8%)	-28·4% (-37·9% to -17·4%)	31·8% (10·9% to 56·6%)
Decile 4	**-0·8% (-6·9% to 5·7%)**	-7·6% (-15·8% to 1·4%)	-33·6% (-45·0% to -20·0%)	39·0% (14·2% to 69·2%)
Decile 5	**18·9% (5·5% to 33·9%)**	17·8% (4·1% to 33·4%)	7·8% (-12·2% to 32·2%)	18·2% (3·7% to 34·7%)
Decile 6	**0·0% (-8·0% to 8·8%)**	0·3% (-8·6% to 10·1%)	7·8% (-8·2% to 26·7%)	-9·2% (-29·8% to 17·4%)
Decile 7	**3·2% (-7·6% to 15·3%)**	1·9% (-8·8% to 13·9%)	-2·9% (-16·1% to 12·4%)	17·6% (-8·3% to 50·8%)
Decile 8	**4·4% (-3·5% to 13·0%)**	-2·1% (-8·8% to 5·1%)	11·1% (-17·9% to 50·5%)	57·0% (12·6% to 118·8%)
Decile 9	**4·2% (-3·5% to 12·4%)**	0·6% (0·1% to 1·1%)	-10·2% (-29·1% to 13·7%)	3·5% (-5·1% to 12·8%)
Decile 10 [Least deprived]	**1·1% (-12·6% to 17·0%)**	-1·1% (-22·3% to 2·6%)	24·6% (3·4% to 50·6%)	7·7% (-15·3% to 37·1%)

Table S13. Change in hospitalisation outcomes associated with alcohol minimum unit pricing legislation, by socio-economic deprivation decile, England uncontrolled model

Deprivation decile	Effect estimate, % (95% confidence interval)			
	**All hospitalisations**	**Hospitalisations for chronic causes**	**Alcoholic liver disease**	**Hospitalisations for acute causes**
Decile 1 [Most deprived]	**9·3% (5·2% to 13·5%)**	10·2% (6·5% to 14·1%)	6·0% (-0·6% to 12·9%)	11·7% (-1·8% to 27·3%)
Decile 2	**7·0% (2·2% to 12·0%)**	6·9% (1·9% to 12·3%)	0·2% (-6·3% to 7·2%)	12·1% (-1·9% to 28·1%)
Decile 3	**11·4% (7·4% to 15·7%)**	9·6% (3·5% to 16·1%)	-1·0% (-3·9% to 2·1%)	17·7% (5·5% to 31·3%)
Decile 4	**13·5% (8·8% to 18·4%)**	13·3% (9·1% to 17·7%)	5·7% (-1·4% to 13·1%)	14·1% (1·9% to 27·8%)
Decile 5	**6·5% (-1·4% to 15·0%)**	12·3% (8·5% to 16·1%)	7·0% (1·2% to 13·2%)	6·6% (-20·9% to 43·6%)
Decile 6	**9·1% (2·1% to 16·5%)**	9·0% (2·1% to 16·3%)	-2·4% (-7·5% to 3·0%)	9·5% (-2·8% to 23·4%)
Decile 7	**7·5% (1·4% to 13·9%)**	1·2% (-6·6% to 9·6%)	-3·6% (-14·0% to 7·9%)	24·1% (1·5% to 51·7%)
Decile 8	**12·1% (5·2% to 19·4%)**	14·8% (9·9% to 20·0%)	14·1% (4·3% to 24·7%)	8·5% (-5·8% to 25·1%)
Decile 9	**5·7% (0·8% to 10·8%)**	4·4% (-1·6% to 10·7%)	6·2% (-8·0% to 22·5%)	6·5% (-4·0% to 18·1%)
Decile 10 [Least deprived]	**2·9% (-0·2% to 6·2%)**	5·0% (1·6% to 8·5%)	11·5% (5·0% to 18·3%)	-2·2% (-14·3% to 11·6%

Data permissions and Statistical Analysis Plan

All Scottish data used in this study were sourced from Public Health Scotland (PHS). English hospitalisations data were sourced from National Health Service (NHS) Digital. An application to request this data was sent to NHS Digital, and following approval, a data sharing agreement was drafted and co-signed by NHS Digital and PHS. English deaths data were sourced from the Secure Research Service (SRS) of the Office of National Statistics (ONS). An application to request this data was sent to ONS, and was subsequently approved (study reference number 1011523). GMAW, DFM, CF, JL, and LG were certified accredited researchers and were the study members with access to all study data.

A pre-specified version of the study's Statistical Analysis Plan was published by Public Health Scotland on 29 November 2022. The Statistical Analysis Plan is available at the link below:

https://publichealthscotland.scot/publications/an-analysis-plan-for-the-evaluation-of-the-impact-of-alcohol-minimum-unit-pricing-on-deaths-and-hospital-admissions-in-scotland/.

We report the following changes to our Statistical Analysis Plan:

- We did not undertake analysis on outcomes defined using any-cause alcohol harm. In our Statistical Analysis Plan, we had indicated this definition may be problematic because there are more coded fields in the English, compared to Scottish, data sources. Upon data retrieval we found that the rate of outcomes were higher in England than in Scotland. This was considered to be due to the increased opportunity to identify outcomes for England, compared to Scotland. These outcomes were therefore not carried forward, as alcohol health harms are understood to be higher in Scotland than England.
- There were several outcomes that we could not estimate effects for. This was due to the low number of outcomes observed. Where this is the case, we have not displayed any estimates.
- We undertook one additional sensitivity analyses for our main deaths and hospitalisations outcomes which involved modelling the difference between the Scottish and English time series to estimate effects, as an alternative to our standard approach which was to add the English time series data as a model covariate in Scottish models.
- Outcomes which are partially attributable to alcohol consumption will be reported by Public Health Scotland in an institutional report released on March 21, 2023.

STROBE statement

The page numbers listed, below, relate to the downloadable PDF version of the manuscript.

	Item No.	Recommendation	Page No.	Relevant text from manuscript
**Title and abstract**	1	(*a*) Indicate the study's design with a commonly used term in the title or the abstract	1	See study **title**
		(*b*) Provide in the abstract an informative and balanced summary of what was done and what was found	1	See study **abstract**
**Introduction**				
Background/rationale	2	Explain the scientific background and rationale for the investigation being reported	1,2	See study **introduction**
Objectives	3	State specific objectives, including any prespecified hypotheses	1,2	See study **introduction**
**Methods**				
Study design	4	Present key elements of study design early in the paper	2,3	See study **methods**
Setting	5	Describe the setting, locations, and relevant dates, including periods of recruitment, exposure, follow-up, and data collection	2,3	See study **methods**
Participants	6	(*a*) *Cohort study*—Give the eligibility criteria, and the sources and methods of selection of participants. Describe methods of follow-up<br>*Case-control study*—Give the eligibility criteria, and the sources and methods of case ascertainment and control selection. Give the rationale for the choice of cases and controls<br>*Cross-sectional study*—Give the eligibility criteria, and the sources and methods of selection of participants	2,3	See study **methods**
		(*b*) *Cohort study*—For matched studies, give matching criteria and number of exposed and unexposed<br>*Case-control study*—For matched studies, give matching criteria and the number of controls per case		Not applicable

Variables	7	Clearly define all outcomes, exposures, predictors, potential confounders, and effect modifiers. Give diagnostic criteria, if applicable	3	See study **methods**
Data sources/ measurement	8*	For each variable of interest, give sources of data and details of methods of assessment (measurement). Describe comparability of assessment methods if there is more than one group	3	See study **methods**
Bias	9	Describe any efforts to address potential sources of bias	3,7,8	See study **methods**/**discussion**
Study size	10	Explain how the study size was arrived at	3	See study **methods**
Quantitative variables	11	Explain how quantitative variables were handled in the analyses. If applicable, describe which groupings were chosen and why	3	See study **methods**
Statistical methods	12	(*a*) Describe all statistical methods, including those used to control for confounding	3,4	See study **methods**
		(*b*) Describe any methods used to examine subgroups and interactions	3,4	See study **methods**
		(*c*) Explain how missing data were addressed		Not applicable
		(*d*) *Cohort study*—If applicable, explain how loss to follow-up was addressed<br>*Case-control study*—If applicable, explain how matching of cases and controls was addressed<br>*Cross-sectional study*—If applicable, describe analytical methods taking account of sampling strategy		Not applicable
		(*e*) Describe any sensitivity analyses	3,4	See study **methods**
**Results**				
Participants	13*	(a) Report numbers of individuals at each stage of study—eg numbers potentially eligible, examined for eligibility, confirmed eligible, included in the study, completing follow-up, and analysed	4,5	See **Figures 1** and **2**
		(b) Give reasons for non-participation at each stage		Not applicable
		(c) Consider use of a flow diagram	3	See **supplementary material**
Descriptive data	14*	(a) Give characteristics of study participants (eg demographic, clinical, social) and information on exposures and potential confounders	4,5	See **Figures 1** and **2**
		(b) Indicate number of participants with missing data for each variable of interest		Not applicable

		(c) *Cohort study*—Summarise follow-up time (eg, average and total amount)		Not applicable
Outcome data	15*	*Cohort study*—Report numbers of outcome events or summary measures over time		Not applicable
		Case-control study—Report numbers in each exposure category, or summary measures of exposure		Not applicable
		Cross-sectional study—Report numbers of outcome events or summary measures	4,5	See **Figures 1** and **2**
Main results	16	(*a*) Give unadjusted estimates and, if applicable, confounder-adjusted estimates and their precision (eg, 95% confidence interval). Make clear which confounders were adjusted for and why they were included	6,7	See **Tables 1**, **2**, **3** and **supplementary material**
		(*b*) Report category boundaries when continuous variables were categorized	3,6	See **study methods/results**
		(*c*) If relevant, consider translating estimates of relative risk into absolute risk for a meaningful time period	6	See **Table 1**

Continued on next page

Other analyses	17	Report other analyses done—eg analyses of subgroups and interactions, and sensitivity analyses	6,7	See **Tables 2** and **3**
**Discussion**				
Key results	18	Summarise key results with reference to study objectives	6,7	See study **discussion**
Limitations	19	Discuss limitations of the study, taking into account sources of potential bias or imprecision. Discuss both direction and magnitude of any potential bias	6,7,8	See study **discussion**
Interpretation	20	Give a cautious overall interpretation of results considering objectives, limitations, multiplicity of analyses, results from similar studies, and other relevant evidence	7,8	See study **discussion**
Generalisability	21	Discuss the generalisability (external validity) of the study results	8	See study **discussion**
**Other information**				
Funding	22	Give the source of funding and the role of the funders for the present study and, if applicable, for the original study on which the present article is based	4	See study **methods**
